# Progress in the Application of Hydrogels in Intervertebral Disc Repair: A Comprehensive Review

**DOI:** 10.1007/s11916-024-01296-6

**Published:** 2024-07-10

**Authors:** Xin Chen, Shaoze Jing, Chenhui Xue, Xiaoming Guan

**Affiliations:** 1 Third Hospital of Shanxi Medical University, Shanxi Bethune Hospital, Shanxi Academy of Medical Sciences, Tongji Shanxi Hospital, Taiyuan, 030032 China; 2 Department of Orthopedics, Shanxi Bethune Hospital, Shanxi Academy of Medical Sciences, Tongji Shanxi Hospital, Third Hospital of Shanxi Medical University, Taiyuan, 030032 China

**Keywords:** Hydrogel, Disc degeneration, Disc regeneration, Tissue engineering, Disc

## Abstract

**Purpose of Review:**

Intervertebral disc degeneration (IVDD) is a common orthopaedic disease and an important cause of lower back pain, which seriously affects the work and life of patients and causes a large economic burden to society. The traditional treatment of IVDD mainly involves early pain relief and late surgical intervention, but it cannot reverse the pathological course of IVDD. Current studies suggest that IVDD is related to the imbalance between the anabolic and catabolic functions of the extracellular matrix (ECM). Anti-inflammatory drugs, bioactive substances, and stem cells have all been shown to improve ECM, but traditional injection methods face short half-life and leakage problems.

**Recent Findings:**

The good biocompatibility and slow-release function of polymer hydrogels are being noticed and explored to combine with drugs or bioactive substances to treat IVDD.

**Summary:**

This paper introduces the pathophysiological mechanism of IVDD, and discusses the advantages, disadvantages and development prospects of hydrogels for the treatment of IVDD, so as to provide guidance for future breakthroughs in the treatment of IVDD.

## Introduction

Low back pain has emerged as a significant global health concern, as indicated by the Global Burden of Disease study report. This condition not only profoundly affects the daily lives and work productivity of individuals but also contributes to a rise in healthcare expenditures, leading to substantial economic and health-related challenges [1]. A substantial percentage, ranging from 26% to 42%, of patients experiencing chronic low back pain attribute their condition to IVDD [2]. Various factors contribute to low back pain, including segmental spinal instability, facet joint degeneration, spinal canal stenosis, nerve root pain, and lumbar disc herniation. IVDD is closely linked to the development of these conditions [3].

The intervertebral disc (IVD) is a fibrous cartilage structure located in the spine, connecting the upper and lower vertebrae. Beyond providing load-bearing support, it allows for a certain degree of spinal curvature to facilitate coordinated human body movement. The IVD comprises three main components: the outer annulus fibrosus (AF), the central core collagen nucleus pulposus (NP), and the cartilaginous endplate (CEP) attached to the upper and lower vertebrae [4]. The AF is a circular structure composed of type I collagen (COL I) and type II collagen (COL II) fibers. It functions to connect the upper and lower vertebrae, limiting and maintaining the NP's position in the central core region. Meanwhile, the NP is rich in COL II, proteoglycan, and water, playing a crucial role in maintaining osmotic pressure and intervertebral height [5, 6]. The IVD is recognized as the largest avascular organ in the body, with the NP positioned over 8 mm away from the nearest capillary network. Consequently, nutrients necessary for NP metabolism are primarily obtained from the external capillary network of the CEP and AF through diffusion, while metabolic waste is discharged through a similar mechanism [7, 8]. In comparison to other IVD components, the NP is situated farther from the capillary network, resulting in higher lactic acid concentration and a lower pH value. The distinctive structural and physiological characteristics of the IVD create an environment characterized by low oxygen levels and a low cell concentration. This low oxygen environment helps mitigate apoptosis of NP cells and IVDD by reducing endoplasmic reticulum stress [8, 9]. However, as age advances, the cell content of the IVD decreases further. The combination of low cell concentration and a low-oxygen environment makes it challenging for the disc to independently recover its original physiological structure after pathological damage. Instead, it relies on slow regeneration to achieve partial recovery [10, 11].

The mechanism underlying IVDD remains incompletely understood, but it appears to be intricately connected to inflammation and an imbalance in ECM synthesis and breakdown. Numerous studies indicate an elevation in inflammatory mediators, such as interleukins (IL-1β, IL-6, IL-8, IL-12), interferon (IFN-γ), tumor necrosis factor-alpha (TNF-α), nitric oxide (NO), and prostaglandin, to varying degrees in degenerated disc tissues [12]. Notably, common inflammatory mediators like TNF-α and IL-6 have been identified as contributors to pain [13]. Recent investigations suggest that an upsurge in inflammatory factors, particularly TNF-α and IL-1β, may promote the apoptotic process of NP cells. This, in turn, accelerates the pathological progression of IVDD, particularly in an IVD environment characterized by low cell density and limited nutrient supply [14]. During IVD degeneration, there is an observed increase in chondrospecific ECM components alongside heightened NP apoptosis [15]. Matrix metalloproteinases (MMPs), aggrecanases/a disintegrin and metalloproteinase with thrombospondin motifs (ADAMTS), and tissue inhibitors of metalloproteinases (TIMPs) are believed to be closely associated with ECM decomposition and anabolism. Additionally, inflammatory factors have been found to upregulate stroma-degrading enzymes (MMPs/ADAMTS), leading to increased ECM degradation and acceleration of IVDD lesion occurrence [16].

**Fig. 1:**
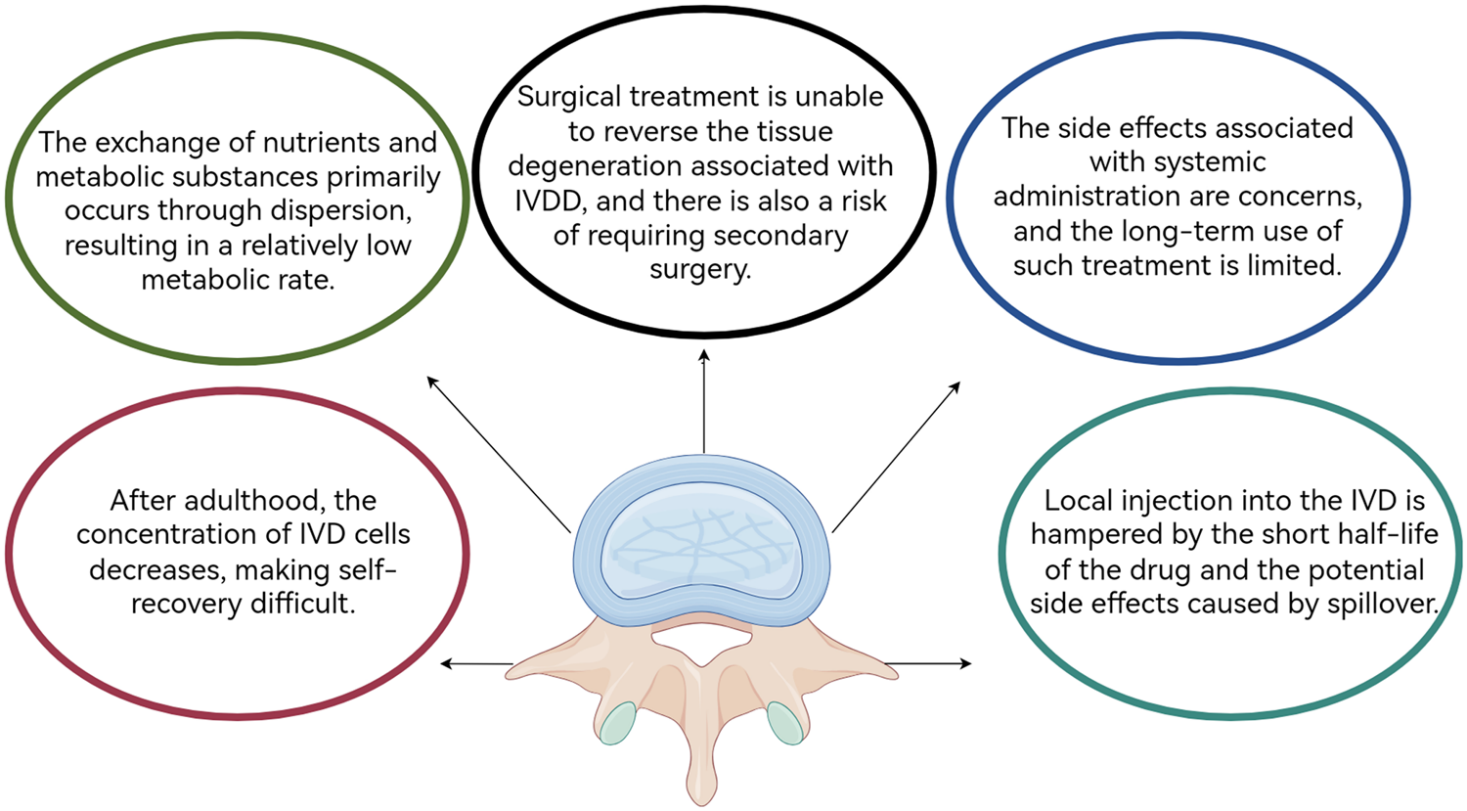
Challenges in the treatment of IVDD by Figdraw

At present, early pain resulting from IVDD is typically addressed through conservative treatment, while patients experiencing significant impacts on work and daily life, along with severe nerve compression, may undergo surgery. Conservative approaches often involve the use of oral non-steroidal anti-inflammatory drugs (NSAIDs) to inhibit cyclooxygenase production and control inflammatory factors, thereby alleviating pain [17]. However, the systemic nature of oral NSAIDs imposes limitations due to potential long-term use and associated gastrointestinal side effects [18]. Moreover, these drugs are incapable of reversing the tissue degeneration characteristic of IVDD [17]. Surgery represents a common clinical intervention for individuals with moderate to advanced IVDD, commonly involving decompression or fusion procedures [19]. Nevertheless, surgical interventions cannot fully restore the original physiological structure of the intervertebral disc. Instead, they may compromise its delicate microenvironment, and there remains a probability of requiring additional surgeries postoperatively [20]. There exists a pressing need for therapeutic methods capable of intervening in the early and intermediate stages of IVDD, aiming to restore the original physiological structure of the intervertebral disc (Fig. 1).

## Potential Treatment: Hydrogels

Hydrogels are polymers featuring a three-dimensional network structure with high water content. The properties of hydrogels exhibit significant variation depending on the specific polymer or crosslinking agent used [6]. In recent years, a variety of smart hydrogels have been developed, including temperature-sensitive, pH-sensitive, light-sensitive, and electric- and magnetic field-sensitive hydrogels. These different types of hydrogels can be selected according to specific application scenarios to meet diverse needs [21]. In the realm of biomedical applications, hydrogels exhibit high biocompatibility, minimizing immune reactions during co-culture with tissues. The three-dimensional network structure of hydrogels serves as a supportive framework for the adhesion of co-cultured cells, and the hydrogel environment, characterized by its high water content, creates an optimal microenvironment for cell survival [22]. Simultaneously, hydrogels possess the ability for the gradual release of loaded substances. Loading cells, bioactive substances, and drugs into hydrogels has demonstrated promising potential in the field of tissue regeneration and repair [23–25]. Hydrogels can be categorized into physical hydrogels and chemical hydrogels based on their different crosslinking mechanisms. Generally, physical hydrogels exhibit strong adaptability and reversibility, while chemical hydrogels possess increased toughness due to the formation of covalent bond crosslinking. Additionally, hydrogels are classified into natural polymer hydrogels and synthetic polymer hydrogels based on their sources. Natural polymer hydrogels generally demonstrate higher biocompatibility, whereas synthetic polymer hydrogels offer greater durability [26]. The following provides a brief overview of several commonly used hydrogel materials in IVDD repair therapy, classified into natural polymer hydrogels and synthetic polymer hydrogels.

## Natural Polymer Hydrogels

### Gelatin

Gelatin is a water-soluble peptide-based polymer derived from the acidic or alkaline hydrolysis of collagen, a protein present in the skin or bones of animals. It exhibits outstanding biocompatibility [27]. Gelatin, rich in hydrophilic (water-absorbing) amino acids such as glycine, proline, and hydroxyproline, forms a three-dimensional network through hydrogen bonding at low temperatures. This process traps water molecules, creating a physical hydrogel. Notably, the gelatin solution transitions back to a soluble state when the temperature rises above 40°C, showcasing thermal reversibility. This characteristic offers a promising avenue for the development of injectable hydrogels [28]. Nevertheless, unmodified hydrogels composed of gelatin exhibit relatively weak mechanical properties. Gelatin, however, contains numerous functional groups that can be coupled or modified using specific targeting ligands to enhance their physical or chemical properties [27]. By incorporating methylacrylyl, the mechanical properties of GelMA can be finely adjusted based on the degree of methacrylate substitution and light intensity. Modified GelMA still maintains excellent biocompatibility [29]. Gelatin inherently contains the RGD (Arg-Gly-Asp) sequence, facilitating increased cell adhesion through integrins and thereby enhancing the cell-carrying therapeutic effect of gelatin-based hydrogels [30]. Moreover, gelatin has been utilized in the development of drug delivery systems for the transportation of drugs, bioactive substances, and cells, contributing to improved therapeutic outcomes [31].

### Collagen

Collagen, an abundantly present protein in the animal body, plays a crucial role as a key component of the extracellular matrix. Its tightly packed triple helix structure imparts a remarkable tensile strength, surpassing that of steel wire with an equivalent cross-section [30]. Similar to gelatin, collagen features the RGD sequence, facilitating increased expression of integrin subunits. This property creates conducive conditions for cell adhesion and establishes a high affinity for adherent cells [32–34]. Simultaneously, hydrogels prepared from collagen have been observed to promote cell differentiation. The activity of cells induces the contraction of collagen, and this mechanical stimulation is deemed to have a positive impact on the differentiation of mesenchymal stem cells/progenitors into osteoblasts. In an experiment by Ivanov et al., hydrogel-supported periodontal ligament stem cells, prepared using collagen, played a role in the differentiation of periodontal ligament stem cells [35]. Hydrogels prepared by coupling collagen with methacrylate demonstrate increased solubility of photoinitiated gelation precursors in neutral and physiological solutions, particularly after the introduction of maleic anhydride [36]. This method has found extensive use in biomedicine due to its low immunogenicity and high biocompatibility. Notably, this high biocompatibility extends to xenogeneic collagen sources as well [37].

### Chitosan

Chitosan, widely distributed in nature, can be obtained through the strong base or chitinase deacetylation of chitin derived from crustaceans, insects, and certain microorganisms. Comprising a linear structure of glucosamine and N-acetylglucosamine [30, 38], chitosan possesses various physiological functions, including biodegradability, biocompatibility, non-toxicity, and bacteriostatic properties. Chitosan-based hydrogels, loaded with antibacterial agents, growth factors, stem cells, and other wound exponents, have been employed to enhance wound healing [39]. Smart hydrogels, synthesized through physical or chemical modification based on chitosan, have found diverse applications in clinical treatments, exhibiting distinct properties in response to pH changes, temperature variations, and light intensity [40]. The development of chitosan as self-healing hydrogels has garnered attention. These hydrogels, created by introducing aldehyde groups and abundant amino groups on chitosan as dynamic cross-linking points to construct imine bonds, showcase promising performance in tissue regeneration. However, the low solubility of chitosan and the nature of imine bonds contribute to poor mechanical properties, limiting their application in areas demanding high-performance mechanical properties [41, 42]. The mechanical properties of chitosan hydrogels through physical crosslinking are insufficient for biological replacement of intervertebral discs. While chemical crosslinking can enhance these properties, concerns arise regarding the cytotoxicity of crosslinking agents.

### Hyaluronic Acid

Hyaluronic acid (HA), a non-sulfated glycosaminoglycan, is highly absorbent and abundant in the vitreous body and brain, playing a crucial role in maintaining normal biological functions. This linear polysaccharide consists of alternating units of D-glucuronic acid and N-acetyl-D-glucosamine linked by β-1,3- and β-1,4-glucoside bonds. As a significant component of the extracellular matrix, HA molecules have garnered considerable attention due to their excellent biocompatibility, natural biological functions, biodegradability, and versatility [39, 43, 44]. Hydrogels based on HA find extensive applications in the biomedical field. When designed to support cells, HA proves beneficial in maintaining cell survival, proliferation, and differentiation. The molecular weight of HA varies from 20,000 to millions of daltons (Da), and its biological function is influenced by the molecular weight. For instance, at higher molecular weights, HA exhibits inhibitory effects on angiogenesis and proliferation [35, 43, 45]. HA molecules exceeding 1 million DA have low solubility due to their high viscosity and HA content. When the molecular weight is less than 50 kDa, HA exhibits increased solubility, allowing for the activity of cultured human mesenchymal stem cells (hMSC) to reach as high as 90% [46]. In a study conducted by Javanmardi et al., HA particles loaded with dexamethasone were prepared to repair sciatic nerve tissue damage. The HA particles demonstrated a sustained release function for the loaded dexamethasone, resulting in a positive therapeutic prognosis [47].

### Synthetic Polymer Hydrogels

Hydrogels made from synthetic polymers typically exhibit superior mechanical properties and durability compared to those composed of natural polymers. However, concerns arise regarding biocompatibility and cytotoxicity.

### Polyethylene Glycol

Polyethylene glycol is a hydrophilic, non-toxic, biocompatible polymer with low immunogenicity, approved by the U.S. Food and Drug Administration (FDA) for human use [48, 49]. Featuring two free hydroxyl groups in its structure, polyethylene glycol is amenable to modification [48]. Studies have explored the use of polyethylene glycol itself or its derivatives in various applications, including wound repair [50], wound closure in eye surgery [51], drug delivery [52], and as an auxiliary agent in imaging, among others [53].

### Poly (N-isopropylacrylamide)

Poly(N-isopropylacrylamide) (PNIPAAM) is an excellent heat-sensitive polymer with a lower critical solution temperature (LCST) of 32°C. This polymer undergoes a hydrophilic-to-hydrophobic transformation at physiological temperatures. Below the LCST, the PNIPAAM molecule is fully stretched and linear, while above the LCST, dehydration and shrinkage occur due to the reversible hydration/dehydration characteristics of the isopropyl side chain [54–56]. Various hydrogels prepared based on PNIPAAM find wide applications in drug delivery, cell delivery, biosensors, and more [55, 57, 58]. However, the inadequate mechanical properties of hydrogels prepared with PNIPAAM alone restrict their application in fields requiring high mechanical properties [59].

Due to the limitations associated with individual materials, current approaches in the treatment of IVDD involve the use of composite hydrogels. These hydrogels combine two or more polymer materials, selected based on their unique properties, to fulfill the mechanical and biocompatibility requirements essential for the treatment process. The development and application of composite hydrogels in IVDD treatment have garnered significant attention from researchers.

**Fig. 2:**
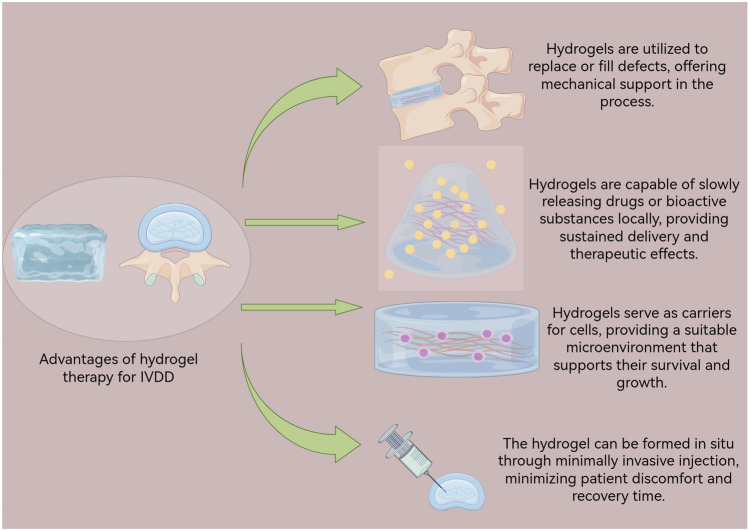
Advantages of hydrogel therapy for IVDD by Figdraw

The injectable hydrogel, characterized by its injectability, good biocompatibility, and loading capacity, is particularly well-suited for the treatment of IVDD. This type of hydrogel offers a viable solution for minimally invasive early-stage IVDD treatment. The injection of the hydrogel into the IVD can fill irregular defective spaces, restoring the normal physiological structure and function of the damaged intervertebral disc. The low shear force during injection helps prevent the death of loaded cells [60]. The slow-release function of hydrogels, coupled with the internal placement characteristics of IVD organs, has inspired researchers to explore hydrogels loaded with drugs or bioactive substances. This approach holds promise for enhancing efficacy, minimizing systemic side effects associated with oral or intravenous pathways, prolonging drug half-life, improving patient compliance, and expanding the treatment window. Ultimately, this can effectively reduce the impact of surgery on patients' lives and mitigate high healthcare costs [24, 61] (Fig. 2).

## Hydrogels are Used to Treat IVDD

We conducted a comprehensive review of recent literature pertaining to the advancements in hydrogel-based treatments for intervertebral disc repair. Our summary focuses on the latest developments in the treatment of IVDD, categorized into four key areas: (1) Simple Hydrogel Injection: Examining the efficacy and progress of direct hydrogel injection in IVDD treatment. (2) Hydrogels Loaded with Bioactive Substances: Summarizing the current state of utilizing hydrogels to support the delivery of bioactive substances for IVDD treatment. (3) Hydrogel-Supported Cells: Providing insights into the progress and applications of hydrogels as a supportive matrix for cells in IVDD treatment. (4) Hydrogel-Supported Drugs: Assessing the latest advancements in utilizing hydrogels as carriers for therapeutic drugs in IVDD treatment.

## Simple Hydrogel Injection

The remarkable mechanical properties of hydrogels make them a promising solution for effectively filling IVDD defects and providing rapid support to restore spinal stability, presenting significant clinical application prospects. In a study by Yang et al., an injectable hydrogel was proposed by crosslinking gelatin and poly(γ-glutamic acid) (γ-PGA) with 1-(3-dimethylaminopropyl)-3-ethyl-carbodiimide hydrochloride (EDC). Tested in a bovine IVD acupuncture model, the hydrogel exhibited good saturation pressure and the ability to maintain IVD pressure. While an increased EDC ratio enhanced these properties, higher EDC concentration raised concerns about increased cytotoxicity [62]. Another study indicated that the toxic effect on cells was not significant when the EDC concentration ranged between 10 and 40 mM. With the elevation of EDC concentration, the hydrogel demonstrated enhanced mechanical strength and resistance to degradation. However, it was observed that an increase in EDC concentration led to a decrease in the secretion of cellular GAG, suggesting a potential hindrance to cell repair [63]. In a separate study, Inoue et al. injected HA hydrogel into an acute injury model of acupuncture-induced IVDD in rabbits to assess its performance. The HA hydrogel was found to maintain intervertebral height, reduce levels of IL-1, IL-6, and TNF-α, and exhibit potential anti-inflammatory effects during the acute phase [64]. Specific investigations revealed that HA hydrogel can interfere with IL-1β, IL-6, and receptor binding, attenuating the acute inflammatory response. It also inhibited the expression of c-Fos and Tac1 at the spinal level in the pain processing neurons of the dorsal horn of the spinal cord, thereby affecting the production of substance P, inhibiting excessive innervation, and reducing the expression of nociceptive receptors TRPV1 and Trk-A. Furthermore, it may promote the regulation of ECM production through the Smad3 family and TGF-β1 [65].

The timing of hydrogel injection significantly affects the treatment prognosis, as emphasized by Liu et al. In their study, hydrogels were prepared by cross-linking HA disulfide with mercaptan modification. Rhesus monkeys were categorized into severe (T1ρ: < 81 ms), moderate (T1ρ: 81~95 ms), and mild (T1ρ: 96~110 ms) degeneration groups based on the MR T1ρ value of the intervertebral disc. The optimal injection time for hydrogels in cases of mild, moderate, and severe IVDD in rhesus monkeys was investigated using T1ρ-magnetic resonance imaging (MRI). Over the course of 12 consecutive months, mild to moderate IVDD demonstrated sustained intervertebral disc strength. However, for cases of severe degeneration, despite effective regeneration of the nucleus pulposus, the prognosis remained poor due to the irreversibility of degeneration, particularly in the facet joints [66].

Lower back pain resulting from IVDD is highly prevalent. Hence, the alleviation of compression and the restoration of IVD stability are deemed advantageous for treatment, contributing to pain relief. Nonetheless, there exists a probability of postoperative neuralgia, and the incapacity to restore the normal physiological structure of the IVD after surgery also impacts the long-term treatment prognosis [67]. Jia et al. proposed a glycerol crosslinked PVA gel for repairing the IVD after NPD. The hydrogel not only restores intervertebral height but also sustains the vitality of nucleus pulposus cells (NPCs) in a pathological state, promoting the anabolism of ECM. This provides a novel approach for the reconstruction of the IVD after clinical NPD [68]. In a clinical study on hydrogels for discogenic low back pain, a favorable safety profile was demonstrated in 20 patients. Among the reported 18 adverse events, most were unrelated to the device or surgery. Two cases involved hydrogel position changes, which were safely addressed post-surgery. At the 6-month follow-up, the median Numerical Pain Rating Scale decreased by 6 points, indicating positive treatment outcomes for disc pain [69]. This underscores the safety and efficacy of hydrogels in the treatment of IVDD.

Simultaneously, to restore the normal IVD structure and stability, the mechanical properties of hydrogels are essential, emphasizing the importance of exploring these properties. In a mechanical analysis of polyvinyl alcohol hydrogels (PVAH), it was observed that as the water content of PVAH increased, the maximum stress of the hydrogels decreased, and the stress relaxation rate increased. While the biomechanical properties of 15% PVAH were more similar to the normal NP in comparison with normal NP mechanical analysis, the facet joint force (FJF) of 20% PVAH was more similar to NP. Intriguingly, the range of motion (ROM) during flexion, extension, lateral bending, and axial torsion did not significantly differ between the two concentrations of hydrogels. While it seems reasonable to consider that 20% PVAH is more suitable for the application in the nucleus pulposus, this study did not break down whether there is a more suitable content group within the 15–20% PVAH hydrogel content [70]. In another approach, cellulose nanofibers (CNFs) were filled with chitosan to create composite hydrogels. Experiments conducted on pig intervertebral discs indicated that an increase in CNFs content resulted in an increased loading capacity of hydrogels. Notably, when the ratio was 3% CHI/0.4% CNF, the mechanical properties of the hydrogels closely resembled those of a healthy IVD [71]. The in-situ gelling of hydrogel offers the advantage of rapid restoration of intervertebral stability, mitigating the impact of unstable factors on the prognosis of continuous stress injury caused by the intervertebral junction.

## Hydrogels are Loaded with Bioactive Substances

The utilization of bioactive substances, supported by hydrogels, in repairing IVDD is a prominent research focus. While bioactive substances are acknowledged for their potential reparative effects on IVDD, the direct injection of these substances into the IVD poses challenges such as a short half-life, adverse effects due to substance spillover, and significant concentration fluctuations. Hydrogels serve as effective carriers, allowing controlled and gradual release of bioactive substances within the IVDD. This innovative approach presents a novel strategy for the injection of bioactive substances in the repair of IVDD.

The dysregulation of ECM synthesis and metabolism in IVDD is considered a causative factor closely associated with the onset and progression of the disease. Therefore, addressing the imbalance in collagen formation and decomposition is a crucial direction to explore in the treatment of hydrogels loaded with bioactive substances. Wang et al. devised an injectable thermosensitive PNIPAAm hydrogel to deliver SHP099, aiming to assess its feasibility in treating IVDD. SHP2, a nonreceptor protein tyrosine phosphatase (Src homologous region 2 contains protein tyrosine phosphatase 2 (SHP2)), exhibits elevated expression in IVDD tissues, inhibiting the expression of sox9, reducing the expression of type II collagen and aggrecan, and accelerating the progression of IVDD tissue destruction. SHP099 effectively suppressed the expression of SHP2, leading to the recovery of sox9 and Col2a1 expression in the modified NP treated with SHP099. In Wang et al.’s rat puncture model, P(NIPAAm)-SHP099 demonstrated its advantages as a hydrogel-supported bioactive substance, extending the half-life of SHP099. The IVDD tissue of the P(NIPAAm)-SHP099 group exhibited significant improvement compared to the hydrogel group and the SHP099 group alone [61].

Inflammation is a pivotal factor in the development of IVDD, and mitigating inflammation levels is crucial for safeguarding the delicate internal environment of the IVD, protecting NPC, and preserving ECM. Utilizing small interfering RNA (siRNA) to target components in inflammatory pathways and alleviate the inflammatory response is an approach to facilitate IVDD repair. Chen et al. developed a multifunctional hydrogel (OG/GCA) derived from oxidized dextran modified with Girard reagent T(OG) and adipic acid dihydrazide grafted cate-chol-coupled gelatin (GCA). A fifth-generation poly(aminamide) modified with phenylboric acid served as a carrier for siRNA targeting the P65/NLRP3 inflammatory signaling pathway. This hydrogel demonstrated sustained siRNA release, effectively blocking the activation of the NLRP3 inflammasome mediated by nuclear factor κ-light chain enhancer (NFκB), thus reducing inflammatory factor levels and improving the IVDD microenvironment. The slow and controlled release of siRNA, facilitated by hydrogel loading, prevented rapid diffusion and degradation [72]. Reactive oxygen species (ROS) contribute to inflammation by activating the STING-NF-κB pathway, playing a role in the pathogenesis of IVDD. Chen et al. developed an injectable hydrogel using aldehyde hyaluronic acid (HA-CHO) and PAMAM to load siRNA complexes, aiming to interfere with abnormal STING signals. The dynamic Schiff base bonds in this system enabled slow and sustained siRNA release. Targeting STING reduced the inflammatory response induced by ROS, contributing to IVDD repair [73]. Kartogenin (KGN) has been explored for its effects on ROS and inflammatory cytokines. Tian et al. loaded KGN into a mercaptocyclodextrin gelatin hydrogel. KGN inhibited ROS, alleviating oxidative stress in rat NPCs, and also suppressed IL-1, contributing to ECM metabolic balance. The protective effects of KGN, however, were diminished in the presence of a nuclear factor erythroid2-related factor 2 (NRF2) inhibitor [24]. In another KGN experiment, IL-4 was introduced to further modulate the inflammatory response. Copolymer PLGA microspheres, composed of lactic acid and glycolic acid, were utilized to facilitate the sustained release of KGN and IL-4. The negatively charged agent sodium methacrylate (SMA) was incorporated into oligo [poly (ethylene glycol) fumarate] (OPF) to form the OPF/SMA hydrogel. The augmentation of negative charge not only fostered cell adhesion, proliferation, and secretion but, more importantly, enhanced the biocompatibility of OPF. Intriguingly, with an increasing SMA ratio, the maximum pressure of OPF/SMA hydrogels decreased from 179 to 109 kPa, while the maximum compressive fracture strain increased from 62 to 78%. PLGA loaded with IL-4, PLGA loaded with KGN, and a mixture with OPF/SMA were employed to produce an IVDD hydrogel. IL-4 was found to promote the polarization of macrophages, inducing the transformation of M0 and M1 into the anti-inflammatory M2 type, thereby reducing the production of pro-inflammatory factors and increasing the production of anti-inflammatory factors (IL-10, IL-13, and TGF-β). KGN also exhibited homing functions for MSCs. In the experiment, the OPF/SMA+IL-4-PLGA hydrogel demonstrated an increased expression of anti-inflammatory cytokines (TNF-α, iNOS) and reduced release of pro-inflammatory cytokines (IL-13). However, the expression of IL-10 did not increase, leading Cheng and colleagues to suggest that IL-10 may be expressed in both M1 and M2. In the rat model simulating IVDD through acupuncture, the therapeutic effect of OPF/SMA loaded with IL-4 and KGN surpassed that of a single drug [74]. MMPs upregulate inflammatory factors and accelerate ECM breakdown, making MMP activity intervention a crucial aspect in improving IVDD prognosis [16]. Xing et al. created an ECM scaffold from pig nucleus pulpoeus decellulator, loading it with Adipose-Derived Stem Cell (ADSC) exosomes to produce an injectable thermosensitive hydrogel dECM@exos. Extracellular vesicles (EVs) inherited from Mesenchymal Stem Cells (MSCs) maintained NPC activity, inhibited apoptosis, and influenced ECM metabolism by decreasing MMP activity. dECM prepared through acellular methods closely resembled the human intervertebral disc structure, presenting a promising clinical prospect [75]. Chai et al. developed a hydrogel using a mixture of HA and batroxase (BTX) to support Platelet-Rich Plasma (PRP) and ferulic acid (FA). PRP, rich in bioactive substances, stimulated NPC proliferation and increased ECM content, while FA, a plant-derived phenolic acid, inhibited the expression of inflammatory factors. This system, by reducing MMPs expression, inhibited ECM decomposition and showed efficacy in inhibiting tissue degeneration in a rat acupuncture IVDD model [76]. Reducing apoptosis in IVD cells during pathological states and maintaining cell activity and number are also considered beneficial for IVDD treatment. Qingxin et al. developed DNA hydrogels using deoxyribonucleate extracted from salmon testicles to encapsulate miRNA mir5590 spherical nucleic acid, with a gold nanoparticle serving as the core, forming miR-5590-SNA@DNAgel. As an ATP-dependent RNA helicase, the up-regulation of DDX5 can lead to mTOR phosphorylation, consequently inhibiting autophagy. While autophagy is known to hinder cell apoptosis, miR-5590 in the hydrogel can down-regulate DDX expression in the IVD, thereby suppressing cell death. Importantly, miR-5590-SNA@DNAgel reduces the expression of ADAMTS and MMP, while promoting COL II expression. This has been demonstrated to be advantageous for NP regeneration [77].

Hydrogels loaded with bioactive substances can significantly impact the number of cells and their activity in IVDD, facilitating ECM production and overall repair of IVDD. Henry et al. devised an injectable biometric hydrogel system, utilizing pullulan microbeads (PMBs) loaded with growth differentiation factor-5 (GDF-5) and transforming growth factor-β1 (TGF-β1) combined with a cellular-based hydrogel. This innovative system demonstrated the sustained release of GDF-5 and TGF-β1 for up to 28 days while preserving their physiological activity [78]. GDF-5 and TGF-β1 have been implicated in promoting the differentiation of ADSC into NPC [79].

## Hydrogel Loaded Cells

**Fig. 3:**
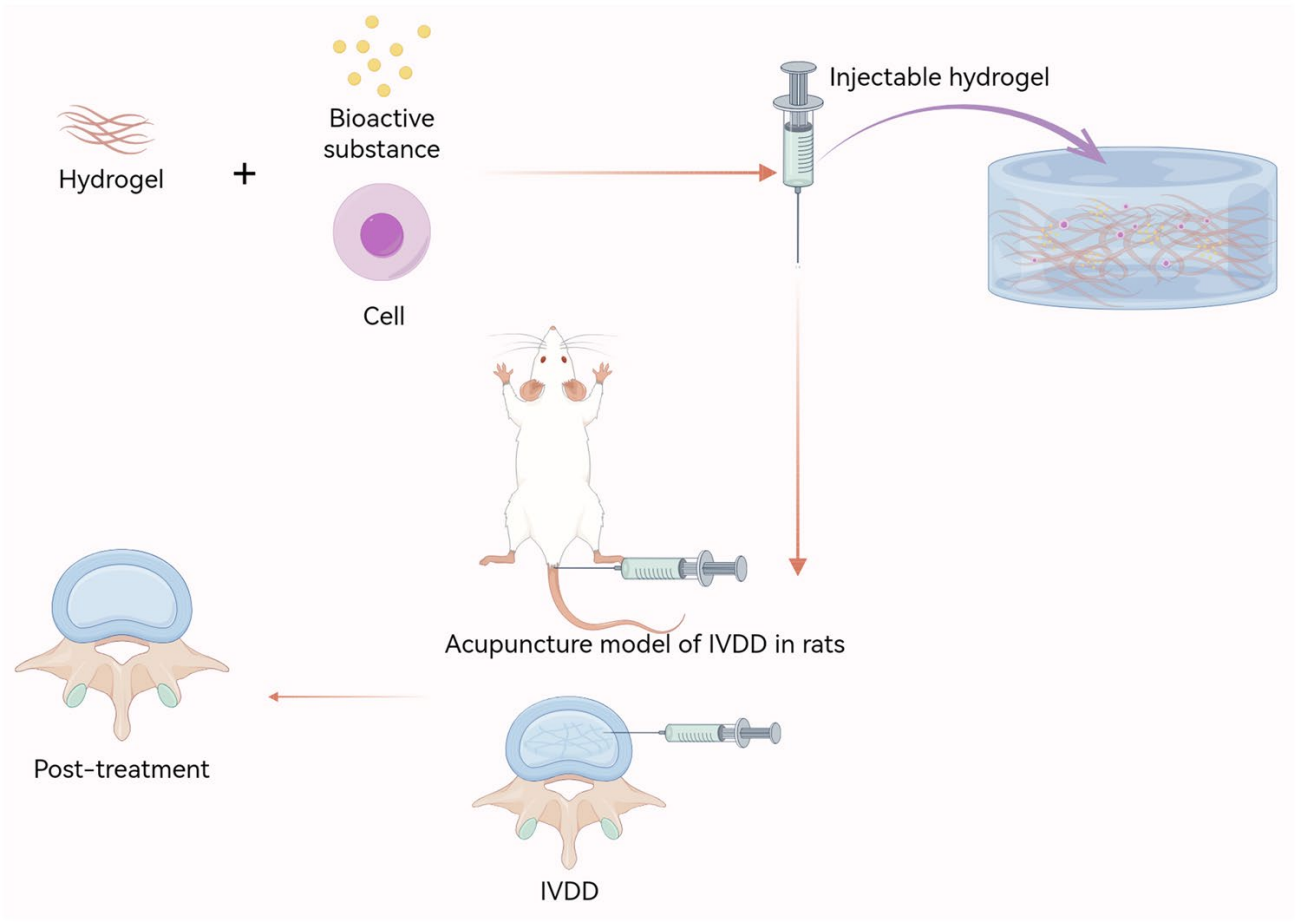
Hydrogel-supported cells and bioactive substances were employed in the treatment of IVDD by Figdraw

The regeneration of IVD has become a research focal point, particularly with the intradiscal injection of cells. Nevertheless, the direct injection of cells into the IVD faces challenges in terms of survival and effective promotion of IVD regeneration, given the adverse microenvironment characterized by IVDD, low nutrient density, and a generally unfavorable cell environment. Moreover, complications such as osteophytes are inevitable after cell overflow. The high biocompatibility of hydrogels offers crucial mechanical support and scaffolding for cell attachment in the context of IVDD, presenting substantial advantages for IVD regeneration. Below is an overview of the cell types selected in various studies (Fig. 3).

NPCs stand out as a common tissue engineering cell in studies related to IVDD repair therapy. In comparison to other cell types, NPC can directly replenish endogenous NPC content without requiring differentiation induction following hydrogel injection. A study conducted by Jiang et al. utilized a hydrogel based on PNIPAAm, silicate ceramics (SC), and sodium alginate (SA) to carry NPC, investigating its potential in IVDD therapy. SC, containing Ca+ and Mg2+ , serves as a crosslinking agent for gel promotion, with Mg2+ released into IVDD promoting a positive response for CD206. This response may facilitate the transformation of macrophages from M1 to M2, playing an anti-inflammatory role, improving the microenvironment, and creating conditions for NPC survival. Western blot analysis revealed that hydrogel-coated NPC enhanced COL II production and reduced MMP13 expression, contributing significantly to IVD regeneration. Direct injection of Mg2+ has a short half-life and risks adverse reactions, but loading it into SC for slow release prolongs the half-life and avoids such reactions [23]. The three-dimensional structure of the hydrogel also plays a crucial role in maintaining the normal phenotype of loaded cells. Wan et al. treated IVDD by transplanting NPCs loaded with a self-assembling peptide hydrogel (SAPH) into the NP of IVDD. In vitro culture showed a decrease in the expression of COL2A1 and Aggrecan (ACAN) during monolayered culture of NPC, while the expression of conventional NP-labeled SOX9 and COL2A1, along with NP-specific labeled KRT8, KRT18, and FOXF1, significantly increased in SAPH. SAPH effectively maintained the NPC phenotype and promoted the secretion of COL II and aggrecan. Additionally, monolayer NPCs produced less COL I and COL II, highlighting the importance of the 3D structure of the hydrogel in maintaining NPC phenotype and normal NP-ECM secretion [80]. Gorapalli et al. conducted a study in which they prepared deacetylated pGlcNAc (DEAC) through deacetylation of N-acetylglucosamine nanofibers (pGlcNAc). DEAC was found to significantly promote NP proteoglycan secretion, exhibiting a 27.7 ± 0.09 times increase compared to the group without DEAC. Notably, hydrogels prepared with DEAC, sulfated pGlcNAc/HDC, and short fiber pGlcNAc/HDC showed higher cell activity and GAG production than hydrogels without DEAC. However, concerns were raised about the hydrogel's significant strength difference from natural NP and its less viscous behavior [81]. Tang et al. addressed this challenge by constructing a double-network hydrogel (GelMA/HA-histidine functionalized hyaluronic acid (HA-His-Mg)) using a microfluidics and UV photocrosslinking method (GHHM). The GHHM microsphere system was employed to deliver NPCs, while Mg2+ maintained cell activity and regulated the ROS-induced inflammatory response associated with IVDD and the worsened microenvironment during IVDD [82]. Hydrogel loading of NPCs and bioactive substances may have advantages for the proliferation and secretion of loaded cells. Benz et al. simulated IVDD in sheep acupuncture by creating a two-component hydrogel of albumin/hyaluronic acid loaded with NPC. This hydrogel was used to compare the effect of hydrogel-loaded cells on IVD regeneration in different segments of the same individual. The intervertebral disc treated with hydrogel-loaded cells exhibited increased COL II mRNA and lower COL I mRNA content, suggesting that this two-component hydrogel is significant for ECM repair of IVDD. Interestingly, sheep have been found to have a tendency to spontaneously recover from damaged intervertebral discs [83], which differs from humans. Benz et al. proposed again that hyaluronic acid-loaded serum albumin was prepared into hydrogels under the action of a polyethylene glycol crosslinking agent, and NPCs were loaded into the hydrogels. Human intervertebral disc cells were cultured separately in vivo and in vitro without hydrogel loading. The presence of hydrogel increased the content of SOX9, speculated to promote the differentiation and maturation of human intervertebral disc cells, and promote the effective deposition of ECM [84]. Francisco et al. employed PEGylated laminin 111 (LM111) by introducing acrylate groups, followed by crosslinking with PEG-diacrylate (PEG-DA) through photopolymerization to prepare a hydrogel. Experimental results indicated that PEG modification of LM111 did not affect its biological function and could still improve the survival ratio of primary NPCs loaded in the hydrogel. However, the small size of the PEG-DA hydrogel formed by photopolymerization may impact metabolite transport and affect NPC activity. Low levels of LM111 may be beneficial for ECM synthesis by increasing the accumulation of GAG and hydroxyproline through elevated lactate concentration [85]. Borrelli et al. utilized EDC to react with the carboxyl group of chondroitin sulfate (CS) to form an unstable amine-reactive ester intermediate. Subsequently, carboxy-activated and functionalized CS (fCS) was produced by adding n-hydroxysuccinimide (NHS). Simultaneously, ECM extracted from bovine intervertebral disc NP was used to prepare an acellular collagen scaffold. The self-crosslinked hydrogel system was generated by the interaction of amines on collagen fibers with fCS. Functionalized (NHS-) CS was employed to form a stable gel structure and enhance the swelling capacity of hydrogels. CS was found to promote the synthesis of sGAGs and selectively inhibit the deposition of collagen in co-culture with pig NPCs. Hydrogels containing CS promoted more COL II deposition (more similar to normal NP tissue) compared to sECM hydrogels without CS, which mainly exhibited COL I deposition. Additionally, it was observed that the DNA content of hydrogel-cultured cells consistently decreased, regardless of the presence of CS, possibly due to the loose fiber network system unable to maintain cell position [86]. In the aforementioned studies on hydrogels loaded with NPCs and loaded with bioactive substances, the addition of bioactive substances was mostly designed to maintain the normal cellular function of NPCs and promote the auxiliary role of NPCs in repairing IVDD. However, challenges such as limited cell sources and premature aging limit the widespread use of NPCs [87].

ADSCs are also frequently employed as tissue engineering cells loaded in hydrogels for IVDD repair. ADSCs, derived from renewable fat tissue, can be supplied in large quantities, reducing the challenges associated with the clinical application of cell-loaded hydrogels. Chen et al. explored the use of photocrosslinked gelatin-hyaluronic acid-methacrylate (GelHA) hydrogels to promote nucleus pulposus differentiation of loaded ADSCs. After GelHA-ADSCs culture, TGF-β1 and TGF-β2 genes were activated, and this effect could be inhibited by integrin αvβ6 neutralizing antibodies, restricting the differentiation of ADSCs into NPCs. It was suggested that GelHA-ADSCs could be activated through the integrin αvβ6-TGF-β1 pathway, contributing to the treatment of IVDD [88]. Yu et al. utilized an acellular nucleus pulposus hydrogel prepared from bovine NP to enhance the biomechanical properties and stability of the hydrogel through cross-linking with genipin, ultimately loading it with ADSCs to evaluate its role in IVD regeneration. As the concentration of genipin increased, the elastic modulus of the hydrogel also increased, but higher concentrations raised concerns about cytotoxicity. Concentrations below 0.02% had no significant effect on cell activity, and the expression levels of Col2 and Sox-9 were higher than other concentrations, while 0.04% concentration caused cytotoxicity leading to cell death. Therefore, it was suggested that 0.02% genipin might be more suitable for developing hydrogel-supported cells for the treatment of IVDD. Despite the negative effect on ADSC activity in the harsh microenvironment of IVDD during long-term culture, improvements in disc height index (DHI), MRI index, and degeneration index were observed in the rat model [89]. The combination of ADSCs and bioactive substances in hydrogel therapy is also a subject of interest. Wang et al. used GelMA loaded with PRP and KGN, in combination with ADSCs, to prepare tissue engineering hydrogels for studying IVD regeneration and the antioxidant effect of KGN. In this study, elevated levels of Col II, ACAN, and cytokeratin 19 were observed in the tissue-engineered hydrogel, and KGN was shown to attenuate the inhibitory effect of ROS on ADSCs proliferation by activating the Nrf2/TXNIP/NLRP3 signaling pathway [90].

hMSCs are commonly utilized in hydrogel-supported cell studies, and their potential in IVDD repair is actively investigated. Hydrogels offer a potential solution to the challenges faced by mesenchymal stem cells, such as control of differentiation. Kumar et al. designed an injectable UV-curable hydrogel, polyhydroxylethyl methacrylate-Co-N-3-aminopropylme thacrylamide grafted with polyamidomine [p(HEMA-co-APMA) g PAA], to coat hMSCs and investigate whether it can promote the differentiation of hMSCs into NP phenotype under hypoxia. The experiment demonstrated an increase in SOX-9, ACAN, and COL II, with a more pronounced increase observed under low oxygen conditions, showcasing the effectiveness of hMSCs for IVDD repair. Needle size and injection speed were found to impact shear force and cell viability during injection, highlighting the importance of these parameters for successful experiments [60]. In a study by Thorpe et al., a pNIPAM DMAc Laponite^®^ heat-sensitive injectable hydrogel delivery system was developed using PNIPAAM, N-dimethylacetamide, and Laponite^®^, characterized by gelation at 37 °C. When cultured in 5% O2 after loading hMSCs, cells could be induced to produce NP-ECM without the need for additional substances, offering advantages in terms of saving experimental costs and promoting clinical use [91]. The heat-sensitive hydrogel loaded with hMSCs produced COL II, aggrecan, and CS in NP, indicating potential for promoting cell differentiation. However, clusters of hMSCs were also observed, making it challenging to determine whether the matrix was secreted by MSCs or endogenous NPCs. The (L-pNIPAM-co-DMAc) hydrogel could be injected directly to fill the gaps of IVDD and restore the mechanical stability of bovine intervertebral discs during the treatment of bovine simulated IVDD, which is crucial for pain treatment [92]. To fully support the growth, differentiation, and proliferation of hMSCs, the addition of bioactive substances to enhance the biological effects of hMSCs has been explored. A hydrogel system supported by BTX composed of HA and PRP was used in bovine whole IVD organ culture in vitro to verify its effect on IVD regeneration. To avoid damage to the AF during hydrogel injection into the NP, the experiment innovatively injected NP from the cartilage end plate. The positive rate of KRT19 expression increased, indicating a potential stimulative effect on cell viability, although significant changes were not observed in the NP markers CA12 and CD24. This may be attributed to the normal IVD used in the experiment rather than IVDD, affecting the content of CA12 and CD24. Hydrogel-loaded hMSCs were observed in bovine IVD and may have a stimulative effect on cell viability in the 0-2 mm range of tissues. However, in the range of 2 to 4 mm, the lower hMSC concentration group had higher histocellular viability [93].

The damage to the AF is a significant factor contributing to symptoms of nerve compression resulting from nucleus pulposus protrusion. Yuan et al. developed a tissue-engineered intervertebral disc (TE-IVD) using a chitosan hydrogel to mimic the NP structure of the IVD. The outer layer of AF was composed of molten poly (butylene succinate-co-butylene terephthalate) (PBST), and the inner layer formed a concentric cylinder through electrospun PBST film winding. The TE-IVD exhibited a compression modulus of 93.54 ± 5.23 MPa in the stress-strain curve, surpassing that of the normal IVD at 42.88 ± 4.36 MPa, meeting the structural demands for IVD. PBST, an aliphatic-aromatic copolyester, provided TE-IVD with excellent mechanical properties and biocompatibility, achieving compressive and tensile strengths of 245 MPa and 418 MPa, respectively, thereby imparting normal AF function and strength to TE-IVD. After implanting AF cells extracted from tendons into the inner layer of AF, TE-IVD was subcutaneously implanted in rats for four weeks before being transferred to rabbit intervertebral disc replacement, demonstrating signs of IVD regeneration in both rats and rabbits [94]. Enhancing AF strength and utilizing AF cells for assistance hold significant promise in addressing the durability and histocompatibility of artificial IVD.

In a comparative study evaluating the regeneration of IVD from nucleus pulposus stem cells (NPSCs) and bone marrow mesenchymal stem cells (BMSCs), Ma et al. utilized a decellularized nucleus pulposus matrix (DNPM) and chitosan to create a hydrogel carrying polylactic acid microspheres containing GDF5. The study revealed that GDF5 significantly elevated the levels of cartilage markers ACAN and COL2A1 in both NPSCs and BMSCs. Interestingly, the chondrogenic effect of GDF5 on NPSCs was found to be stronger than on BMSCs. Moreover, hydrogels loaded with NPSCs exhibited excellent potential for IVD regeneration in animal experiments, highlighting their promising role in regenerative therapies [95].

Luo et al. developed ECM-Gels hydrogels utilizing the extracellular matrix from rib cartilage and collagen enriched with various cytokines. In addition, lentivirus engineering was employed to create cartilage endplate stem cells (CESCs) expressing exogenous sphingosine kinase 2 (Sphk2). Loaded into ECM-Gels, CESCs were injected into the IVD through the vicinity of the cartilage endplate to minimize AF damage. Sphk2 plays a crucial role in IVD regeneration by regulating autophagy through the PI3K/AKT signaling pathway [96]. Frith et al. developed an injectable hydrogel system based on horseradish peroxidase (HRP) and H2O2 crosslinked tyramine-functionalized hyaluronic acid (HATYR) and 3-4-hydroxyphenylpropionic acid-functionalized PEG (PEGHPA). Pentene polysulfide (PPS) was incorporated to assess the differentiation and growth of mesenchymal precursor cells (MPCs) in hydrogels and the therapeutic potential for IVDD. The addition of PPS resulted in increased deposition of GAG and COL II around cells, indicating a promotion of matrix formation. The HA/PEG hydrogel system loaded with PPS exhibited good biocompatibility when subcutaneously injected into rats, with no significant immune response observed. The system was capable of promoting the differentiation of MPCs towards ECM generation without the need for additional pro-differentiation cytokines such as TGF-β, demonstrating high application potential [97].

Dynamic observation of the position of hydrogels after IVDD implantation is crucial for ensuring the efficacy and safety of hydrogel therapy. However, the highly water-containing nature of hydrogels poses challenges for imaging technologies. In a study by Gullbrand SE et al., zirconia nanoparticles were incorporated into hydrogels. Co-culture with NPC and mesenchymal stem cells (MSCs) revealed no adverse effects on cell viability. Interestingly, the GAG content in NP hydrogels containing zirconia nanoparticles was significantly reduced, potentially promoting the synthesis of type II collagen in MSC [98]. This innovation allows for real-time monitoring of hydrogel positions post-injection into the intervertebral disc, aiding in determining their location and offering guidance for subsequent interventions if needed (Table 1).

**Table 1:** Hydrogels are used to treat IVDD cells

Cell type	Cell origin	Goal	Role in the treatment of IVDD	Refer to
NPCs	Cows, rats, sheep	NPC, ECM	Replenish the reduced NPCs in IVDD and regenerate the nucleus pulposus matrix.	[23, 80–83]
CESC	Young mouse	Exosome	Release exosomes that carry specific cell-active substances and regulate cell activity.	[96]
ADSCs	Rat	NPC, ECM	NPCs-like differentiation promotes the production of ECM.	[88–90, 97]
MPCs	Human	ECM	Chondroid differentiation promotes ECM production.	[97]
hMSCs	Human	NPC, ECM	Promote differentiation into NPCs and promote the production of ECM.	[60, 91–93]
AF cell	Rabbit	AF matrix	Promote differentiation into NPCs and promote the production of ECM.	[94]
NPSCs	Rat	NPC, ECM	Promote differentiation into NPCs and promote the production of ECM.	[95]
BMSCs	Rat	NPC, ECM	Promote differentiation into NPCs and promote the production of ECM.	[95]

Under the fragile microenvironment, low nutrition, low oxygen, and low cell concentration of IVDD, it is difficult for the IVD to complete the regeneration and repair of IVD by itself. Hydrogel-loaded cells to improve the microenvironment in IVDD and loaded bioactive substances to maintain and improve the cells' ability to proliferate and differentiate, providing a potential solution for IVDD repair.

## Hydrogel Loaded Drug

Drug therapy plays a crucial role in the repair of IVDD. Local injection of hydrogel-loaded drugs into the intervertebral disc provides a targeted approach to IVDD treatment. The selection of drugs primarily aims to reduce cell apoptosis, decrease inflammatory factors, alleviate pain, and improve the local environment. The slow-release function of hydrogels allows for sustained drug release, avoiding the systemic side effects associated with systemic drug administration. Additionally, it addresses issues such as drug leakage and the unsustainability of drug concentration seen with direct intra-disc injections. In a study by Pan et al., gefitinib demonstrated a protective effect on rat NPCs. This effect was found to be mediated through the EGFR-autophagy axis, as evidenced by the loss of protection upon knockdown of the autophagy-associated gene 5 (Atg5). The thermoresponsive AHA-g-PNIPAAm copolymer hydrogel loaded with gefitinib further showed promise in reducing NP tissue deterioration and maintaining disc stability in a rat model of intervertebral disc acupuncture when compared to a treatment group without gefitinib loading. A 5-year follow-up study on five patients treated with gefitinib for small cell lung cancer also demonstrated significant improvement in their initial IVDD condition [99]. NSAIDs are commonly utilized as anti-inflammatory pain relievers. Celecoxib, a selective cyclooxygenase-2 inhibitor, is loaded into a chitosan hydrogel to reduce the production of prostaglandins, achieving anti-inflammatory and pain-relieving effects. Hydrogels not only provide structural support but also create an improved microenvironment for NPC, thereby slowing the progression of IVDD [25]. High expression of vascular endothelial growth factor (VEGF) is observed in human and rat IVDD. The use of a thermosensitive injectable poly(lactide-co-glycolide)-block-poly (ethylene glycol)-block-poly(lactide-co-glycolide) (PLGA-PEG-PLGA) hydrogel loaded with bevacizumab for IVDD treatment has been investigated. Bevacizumab, by inhibiting VEGF expression, is shown to reduce matrix metalloproteinase 3 (MMP3) levels, promoting the synthesis of COL II. The thermosensitive hydrogel rapidly forms in situ seconds after injection, preventing drug leakage [100]. Yang et al. have developed an injectable hydrogel named PBNPs@OBG (Prussian blue nanoparticle @ oxidized hyaluronic acid/borax/gelatin). This hydrogel delays the expression of MMPs, reduces ECM degradation, and continuously releases Prussian blue nanoparticles (PBNPs). It is designed to remove inflammatory factors from the IVDD environment, protecting NPC from abnormal activity [101].

## Conclusion and Prospect

Notochordal cells are recognized as precursor cells of the NPC and play a crucial role in the development and maturation of the IVD. The loss of notochordal cells is believed to precede IVDD [102]. While rats are frequently employed in IVDD metamorphosis studies, it's important to note that rats may retain a few notochordal cells until 16 months of age. Notochordal cells have also been identified in the intervertebral discs of 12-month-old rabbits and 3-month-old pigs. Conversely, no notochordal cells have been detected in the intervertebral discs of 2-year-old dogs and 4-year-old sheep [103]. Humans typically experience the loss of notochordal cells during adolescence. Consequently, outcomes obtained in animal IVDD models using hydrogel therapy may yield more favorable results due to the presence of notochordal cells compared to the outcomes observed in human IVDD therapy. This discrepancy underscores the importance of considering species-specific characteristics and developmental timelines when extrapolating findings from animal studies to human conditions.

The remarkable biocompatibility and biomechanical properties of hydrogels hold great promise for their application in the regeneration and repair of IVD. Employing hydrogels to deliver drugs and bioactive substances into the IVD not only addresses the challenging microenvironment within IVDD but also rapidly restores the stability of spinal joints and fosters IVD regeneration. The introduction of hydrogel-supported cells into the context of IVDD further amplifies this regenerative effect, presenting a novel and enhanced treatment approach for clinical practice. This innovative strategy leverages the unique properties of hydrogels to potentially revolutionize the landscape of IVD therapy, offering a path towards effective regeneration and improved outcomes for individuals experiencing intervertebral disc degeneration.

## Data Availability

No datasets were generated or analysed during the current study.

## Abbreviations

IVDD: Intervertebral disc degeneration
ECM: Extracellular matrix
IVD: Intervertebral disc
AF: Annulus fibrosus
NP: Nucleus pulposus
CEP: Cartilaginous endplate
COL I: Type I collagen
COL II: Type II collagen
NO: Nitric oxide
MMPs: Matrix metalloproteinases
ADAMTS: Aggrecanases/a disintegrin metalloproteinase with thrombospondin motifs
TIMPs: Tissue inhibitors of metalloproteinases
RGD: (Arg-Gly-Asp) sequence
HA: Hya-luronic acid
DA: Daltons
hMSC: Human mesenchymal stem cells
FDA: Food and Drug Administration
PNIPAAM: Poly(N-isopropylacrylamide)
LCST: Lower critical solution temperature
EDC: 1-(3-Dimethylaminopropyl)-3-ethyl-carbodiimide
γ-PGA: Poly(γ-glutamic acid)
MRI: Tρmagnetic resonance imaging
NPD: Nucleus pulposus discectomy
NPC: Nucleus pulposus cells
PVAH: Polyvinyl alcohol hydrogels
FJF: Facet joint force
ROM: Range of motion
CNFs: Cellulose nanofibers
SHP2: Src homologous region 2 contains protein tyrosine phosphatase 2
OG: Oxidized dextran modified with Girard reagent T
GCA: Grafted catechol-coupled gelatin
ROS: Reactive oxygen species
KGN: Kartogenin
NRF2: Nuclear factor erythroid2-related factor 2
SMA: Sodium methacrylate
OPF: Oligo [poly(ethylene glycol) fumarate]
ADSC: Adipose-derived stem cell
EVs: Extracellular vesicles
MSCs: Mesenchymal stem cells
BTX: Batroxase
FA: Ferulic acid
PRP: Platelet-rich plasma
GDF-5: Growth differentiation factor-5
PMBs: Pullulan microbeads
SC: Silicate ceramics
SA: Sodium alginate
SAPH: Self-assembling peptide hydrogel
ACAN: Aggrecan
DEAC: Deacetylated pGlcNAc
pGlcNAc: N-acetylglucosamine nanofibers
GelMA: Gelatin methacrylamide
LM111: Laminin 111
PEG-DA: PEG-diacrylate
CS: Chondroitin sulfate
fCS: Functionalized CS
NHS: N-hydroxysuccinimide
GelHA: Gelatin-hyaluronic acid-methacrylate
DHI: Disc height index
TE-IVD: Tissue-engineered intervertebral disc
PBST: Poly (butylene succin-ate-co-butylene terephthalate)
NPSCs: Nucleus pulposus stem cells
BMSCs: Bone marrow mesenchymal stem cells
DNPM: Decellularized nucleus pulposus matrix
CESCs: Cartilage endplate stem cells
Sphk2: Sphingosine kinase 2
HRP: Horseradish peroxidase
PEGHPA: 3-4-Hydroxyphenylpropionic acid-functionalized PEG
PPS: Pentene polysulfide
GAG: Glycosaminoglycans
MPCs: Mesenchymal precursor cells
MSC: Mesenchymal stem cells
NSAIDs: Non-steroidal anti-inflammatory drugs
VEGF: Vascular endothelial growth factor
PBNPs: Prussian blue nanoparticles
